# Optimizing Tuberculosis Incidence Prediction: A Systematic Review of Hybrid Modeling Approaches and Machine Learning Techniques

**DOI:** 10.1002/puh2.70391

**Published:** 2026-09-29

**Authors:** Dip Bahadur Singh, Bipindra Pandey, Bibek Giri, Yashoda Dangi, Sagun Kharel, Roshan Kumar Mahato

**Affiliations:** ^1^ Department of Health Informatics School of Engineering Kathmandu University Dhulikhel Nepal; ^2^ Department of Pharmacy Madan Bhandari Academy of Health Sciences, Hetauda, Bagamati Province Hetauda Nepal; ^3^ College of Global AI Convergence, Seoul Christian University Seoul South Korea; ^4^ Valley College of Technical Sciences Purbanchal University Kathmandu Nepal; ^5^ Department of Nursing Madan Bhandari Academy of Health Sciences, Hetauda, Bagamati Province Nepal; ^6^ Faculty of Public Health Khon Kaen University Khon Kaen Thailand

**Keywords:** ARIMA‐LSTM, forecasting, hybrid models, low‐ and middle‐income countries (LMICs), narrative synthesis, public health, tuberculosis

## Abstract

**Background:**

Tuberculosis (TB) remains a significant global health problem, particularly in low‐ and middle‐income countries (LMICs), where accurate prediction of TB incidence is crucial for effective public health interventions.

**Objective:**

This review assessed and compared statistical, machine learning (ML), and hybrid models for forecasting TB incidence. We also propose a framework to guide model selection based on resources and data.

**Methods:**

A systematic review followed PRISMA 2020 guidelines with protocol in PROSPERO (CRD42025633162). We searched five databases for studies from 2013 to 2024. Studies using TB incidence forecasting models with quantitative metrics were eligible. Due to heterogeneity (*I* ^2^ = 99.89%), we conducted narrative synthesis. Subgroup analyses examined geographic variation, with risk of bias assessed using ROBIS tool. Publication bias was evaluated through funnel plots and Egger's test.

**Results:**

We included 29 studies. Hybrid models, particularly ARIMA‐LSTM, demonstrated superior forecasting accuracy in most contexts, with reported mean absolute percentage error (MAPE) values ranging from 4.06% to 11.2%. In contrast, standalone ML models showed MAPE between 8.88% and 26.93%, and statistical models ranged from 3.77% to 29.37%. However, only 36% of studies validated models using external datasets, and fewer than 30% incorporated exogenous variables. Subgroup analysis revealed geographic disparity: Within China, single models slightly outperformed hybrids (mean MAPE 8.76% vs. 12.95%), whereas outside China hybrids were markedly better (5.42% vs. 28.24%). Publication bias was suggested by funnel plot asymmetry (Egger's test *p* = 0.22). Risk of bias was moderate to high in most studies, primarily due to lack of external validation.

**Conclusion:**

Hybrid models, especially ARIMA‐LSTM, often provide accurate TB incidence forecasts, but performance is highly context‐dependent. Future research must prioritize external validation, integration of exogenous variables, and model interpretability. The proposed evidence‐based framework can assist model selection in resource‐limited LMIC settings.

## Graphical abstract

This systematic review and meta‐regression compared the performance of statistical, machine learning, and hybrid models in forecasting tuberculosis (TB) incidence. The study followed PRISMA 2020 guidelines and included studies published between 2010 and 2024. Hybrid models, particularly ARIMA‐LSTM, demonstrated superior forecasting accuracy compared to standalone statistical and machine learning models. However, only 36% of the studies validated their models using external datasets, and fewer than 30% incorporated exogenous factors, limiting the models' generalizability. Substantial heterogeneity and publication bias were observed across the included studies. The review proposes an evidence‐based model selection framework considering data characteristics, model strengths, and interpretability. Future research should focus on external validation, integration of exogenous variables, and the use of explainable AI methods to improve model transparency and applicability in public health decision‐making.

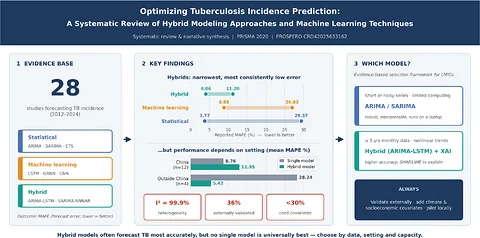

## 1 Introduction

The study in Taiwan (2004–2008) applied a Poisson regression model, identifying temperature, seasonality, age, and aboriginal status as key predictors of TB trends, with the highest incidence in the eastern regions, emphasizing the potential of broader risk factor integration for improved forecasting [1]. In Iran, SARIMA modeling revealed seasonal TB peaks in spring and summer, forecasting 12 cases per 100,000 in 2015, although limited by provincial scope [2]. Another Iranian study using an SARIMA(0,1,1)(0,1,1)_12_ model predicted 16.75 cases per 100,000 in 2014 with good fit but highlighted the need to incorporate external variables such as HIV and drug resistance for enhanced prediction [3]. GIS and statistical modeling across 337 Chinese municipalities demonstrated strong associations between TB prevalence and environmental, geographic, and social factors, with geographically weighted regression (GWR) explaining 52.6% of local variation [4]. In Xinjiang, China, ARIMA and ARIMA‐ARCH models were compared, with ARIMA‐ARCH achieving superior forecasting accuracy by accounting for data variability, suggesting a future model and geographic expansion for broader application [5].

This review of forecasting studies found that in Singapore, TB incidence among non‐residents declined by 3% annually, with SARIMA models effectively capturing trends despite limited socioeconomic data [6]. In Jiangsu, China, an ARIMA‐NAR hybrid model achieved higher accuracy than ARIMA alone by modeling linear and nonlinear patterns, with recommendations to include more variables for broader applicability [7]. Compartmental models in Victoria and Amsterdam showed two‐stage latency structures that better reflected TB dynamics, suggesting recalibration using real‐world data and expansion to high‐incidence regions [8]. In Lianyungang, China, an ARIMA‐GRNN hybrid improved short‐term TB forecasts over ARIMA alone [9], whereas an SARIMA‐NARNNX hybrid provided superior long‐term forecasts in China but indicated a need for external variables [10]. An SARIMA‐NNNAR hybrid in Qinghai outperformed individual models by effectively combining linear and nonlinear patterns [11]. In India, ARIMA modeling during COVID‐19 in Churu revealed a sharp decline in TB detection, highlighting the impact of the pandemic on public health surveillance [12]. Exponential smoothing accurately predicted decreasing TB trends in Henan, China, aligning with WHO targets, but emphasized the need for model updating [13]. ARIMA models forecasting monthly TB incidence in Anhui achieved high accuracy (average error 1.91%) during the COVID‐19 era [14]. Finally, an SARIMA(3,1,0)(0,1,1)_12_ model forecasted MDR‐TB trends in Guizhou, China, identifying a 62.1% case increase from 2019 to 2020 and seasonal clustering, recommending the inclusion of socioeconomic and environmental factors for improved forecasts [15].

This study found that integrating meteorological factors significantly enhanced TB prediction accuracy in three eastern Chinese cities, with the ARIMAX model outperforming ARIMA and RNN models [16]. A SARIMA((2),0,(2))(0,1,0)_12_ model accurately forecasted TB incidence in Guangxi, China, although it lacked integration of external factors such as HIV or MDR‐TB, limiting broader applicability [17]. A CEEMDAN‐TCN hybrid model demonstrated superior time‐series forecasting performance compared to LSTM and ARIMA on artificial and real datasets, despite requiring substantial computational resources [18]. In Taiwan (2005–2020), the SARIMA‐ETS hybrid model provided the best short‐term TB predictions, whereas ETS alone was more effective for long‐term forecasts, supporting public health planning [19]. A global review of over 100 studies showed that ARIMA‐based hybrid models significantly outperformed standalone ARIMA in environmental forecasting, particularly for pollutant data [20]. A comparison of ARIMA, GM(1,1), and LSTM models for TB incidence prediction in China (2018–2020) revealed LSTM's superior ability to capture complex nonlinear trends, suggesting the need to incorporate socioeconomic factors for improved predictions [21]. A smoothed CNN (S‐CNN) model outperformed CNN, MLP, and LSTM for daily visitor data forecasting, achieving the lowest MSE and mean absolute percentage error (MAPE) and highlighting the potential of hybrid models across diverse datasets [22]. Lastly, the EDAM deep learning system developed for hepatic echinococcosis detection using CT images surpassed radiologists’ diagnostic accuracy, although future improvements necessitate a larger, balanced dataset [23].

Multivariate LSTM and ARIMA‐LSTM hybrid models improved TB incidence prediction in Liaoning, China, with the three‐step ARIMA‐LSTM model showing strong medium‐ to long‐term forecasting accuracy [24]. Similarly, the hybrid EMD‐ARMA‐LSTM model enhances TB prediction by integrating multiple techniques with retrospective Chinese data [25]. In Bogotá (2007–2020), ARIMA(7,0,9) outperformed NAR, LSTM, and RBF networks, supporting the recommendation for hybrid models and the inclusion of external covariates [26]. SARIMA models effectively captured PTB and EPTB seasonality in Medea, Algeria (2008–2017) but lacked socioeconomic and environmental factors, suggesting future model refinement [27]. In COVID‐19 forecasting, ARIMA outperformed others in India, whereas ARIMA‐GRNN was superior in the United States, emphasizing the need for region‐specific prediction strategies [28]. National data from Ghana (2016–2020) revealed that COVID‐19 caused sharp declines in TB notifications (−32.7%) and HIV testing (−40.3%), with a transient rise in ART initiation, highlighting pandemic‐induced disruptions in essential health services [29].

### 1.1 Novelty of Statement

This study is the first to systematically review and narratively synthesize the evidence on statistical, machine learning (ML), and hybrid models for TB incidence forecasting across diverse settings. By examining study characteristics, geographic variations, and methodological quality, we identify key gaps and propose an evidence‐based model selection framework tailored to low‐ and middle‐income countries (LMICs).

### 1.2 Objectives of the Study

The study conducted a systematic review and narrative synthesis to evaluate and compare the predictive performance of statistical, ML, and hybrid models in tuberculosis (TB) incidence forecasting and to propose an evidence‐based model selection framework suitable for diverse resource settings.

## 2 Methodology

### 2.1 Study Design

This systematic review and narrative synthesis aimed to evaluate and compare the performance of predictive models in forecasting TB incidence. This study followed the PRISMA 2020 guidelines for transparency and rigor. The review protocol was registered with PROSPERO (CRD42025633162). Due to substantial methodological and clinical heterogeneity among studies, a meta‐analysis was deemed inappropriate; instead, a narrative synthesis was conducted to summarize findings, explore patterns, and identify contextual factors influencing model performance.

### 2.2 Search Strategy

A thorough search was conducted across PubMed, Embase, Web of Science, and Scopus for studies published between 2010 and 2024. MeSH terms and free‐text keywords, such as forecasting, prediction, statistical models, and ML, were used to ensure a broad search. Gray literature and manual reference screening minimized publication bias. The search terms and strategies used are detailed in Supplementary Table 1.

### 2.3 Study Selection Criteria

Original studies that used predictive models (statistical, ML, and hybrid) for TB forecasting and reporting evaluation metrics (MAPE, root mean square error [RMSE], or mean absolute error [MAE]) were included. Studies in English with clear methodologies and sufficient data for comparison were selected. Duplicates, non‐original studies, and studies without quantitative metrics were excluded. Two independent reviewers screened the studies and resolved discrepancies through discussion. The PRISMA flow diagram (Figure 1) illustrates the selection process.

**FIGURE 1:**
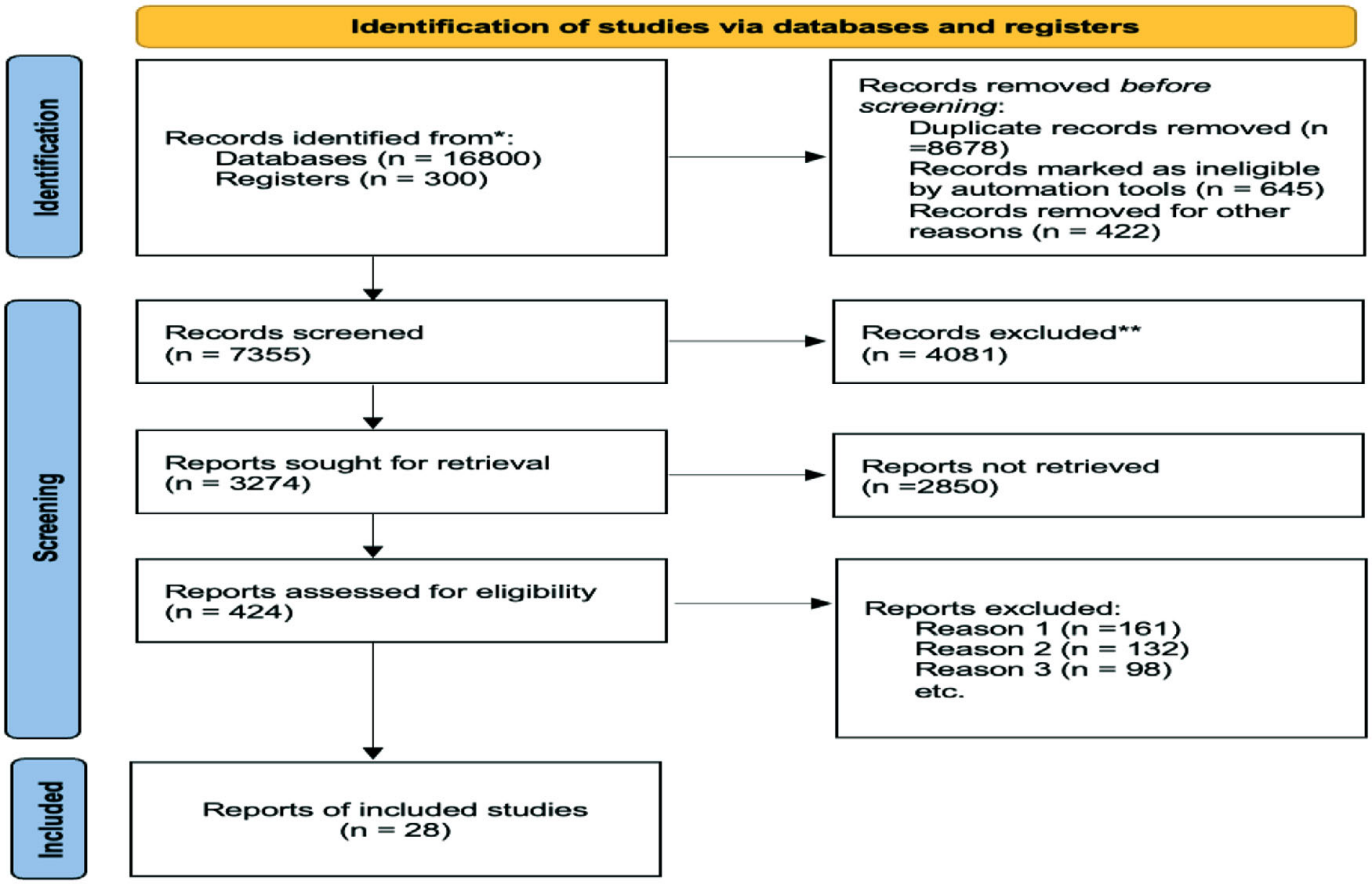
PRISMA 2020 flow diagram.

### 2.4 Data Extraction and Management

Data were independently extracted by two reviewers using a standardized form. The extracted variables included authors, year, model type, sample size, performance metrics, and key findings. Data management was performed using Python (version 3.12) with Pandas and NumPy to ensure consistency. Discrepancies were resolved through discussion.

### 2.5 Evidence‐Based Model Selection Framework

An evidence‐based framework was developed based on the synthesized findings, providing guidelines for selecting the best model for TB forecasting under various conditions. The framework considers data characteristics, model strengths, and computational constraints and includes practical implementation considerations for LMICs.

### 2.6 Data Synthesis and Analysis

Given the high heterogeneity expected, a narrative synthesis was planned. We summarized study characteristics, model types, performance metrics, and key findings in tables and text. Subgroup analyses were conducted by geographic region (China vs. non‐China) and model category (statistical, ML, hybrid). Heterogeneity was quantified using the *I* ^2^ statistic from a random‐effects meta‐analysis of MAPE values (DerSimonian–Laird estimator) performed in R (version 4.3.1, metafor package) to confirm the degree of variability, but pooled estimates are not reported due to extreme heterogeneity. Sensitivity analysis used log‐transformed MAPE to assess skewness. Publication bias was assessed visually using funnel plots and statistically with Egger's test, with standard errors of MAPE approximated as SD/√*n* from reported data.

### 2.7 Risk of Bias Assessment

The adapted ROBIS tool assessed bias in four domains: eligibility criteria, study selection, data collection, and data synthesis. We adapted ROBIS for primary predictive modeling studies by modifying domain signaling questions to focus on model development and validation practices (e.g., external validation, handling of overfitting, and reporting of performance metrics). Studies were categorized as low‐, moderate‐, or high‐risk on the basis of quality. Low‐risk studies used external validation, large sample sizes, and robust methods, whereas high‐risk studies lacked validation and had small sample sizes.

### 2.8 Heterogeneity Analysis

Heterogeneity was assessed using the *Q*‐statistic and *I* ^2^ statistic. An *I* ^2^ > 75% indicated significant heterogeneity, confirming the decision to forego meta‐analysis and proceed with narrative synthesis.

## 3 Results

These findings suggest that although hybrid models can enhance TB forecasting accuracy, their effectiveness is highly context‐dependent, particularly influenced by data quality, regional epidemiological factors, and modeling practices. Improving model generalizability requires integrating socioeconomic and environmental covariates, rigorous external validation, and adopting standardized evaluation frameworks across diverse settings.

### 3.1 Study Characteristics

A comprehensive summary of the included studies is provided in Table 1. These studies, published between 2012 and 2024, primarily focused on forecasting TB incidence using statistical models (e.g., ARIMA, SARIMA), ML techniques (e.g., LSTM, CNN), and hybrid frameworks (e.g., ARIMA‐LSTM, SARIMA‐NNNAR). The sample sizes varied substantially, ranging from 365 to over 3 million observations. Forecasting performance is most commonly assessed using MAPE, RMSE, and MAE as presented in Table 1.

**TABLE 1:** Study characteristics.

Authors	Published year	Country/Region	Study design	Major outcomes	Main results	Evaluation metrics
Zhao et al.	2023	China	EMD‐ARMA‐LSTM	TB incidence prediction	Improved prediction over EMD‐SARIMA	MSE: 21.7%, MAE: 10.6%, RMSE: 11.5%, MAPE: 11.2%
Yang et al.	2022	China	ARIMA‐LSTM hybrid	TB forecasting	Three‐step ARIMA‐LSTM better for long‐term	Error reduction: 13.5%–36.8%
Sun et al.	2015	China	GIS/Statistical modeling	TB risk factors identification	Environmental and social factors linked to TB	*R* ^2^: 0.439, GWR *R* ^2^: 0.526
Wang et al.	2020	China	SARIMA‐NNNAR hybrid	TB incidence forecast	SARIMA‐NNNAR hybrid outperformed SARIMA	MAE: 0.424/0.803, RMSE: 0.564/0.979, MAPE: 5.610%/7.649%
Zhang et al.	2020	China	Exponential smoothing	TB trend prediction	TB registrations decreased towards WHO targets	MAE: 0.334, RMSE: 0.452, MAPE: 5.527%
Guo et al.	2023	China	CEEMDAN‐TCN hybrid	Ionospheric/air quality prediction	CEEMDAN‐TCN outperformed LSTM and ARIMA	Artificial data: RMSE: 0.4404, MAE: 0.3298, MAPE: 5.0024%, *R* ^2^: 0.9924; TEC data: RMSE: 1.84, MAE: 1.38, MAPE: 9.52%, *R* ^2^: 0.94
Li et al.	2019	China	ARIMA‐GRNN hybrid	Short‐term TB incidence prediction	ARIMA‐GRNN hybrid better than ARIMA	ARIMA: RMSE: 0.2805, MAE: 0.2261, MAPE: 8.8797%; ARIMA‐GRNN: RMSE: 0.2553, MAE: 0.1519, MAPE: 5.7222%
Chen et al.	2022	China	ARIMA model	TB incidence during COVID‐19	High‐accuracy forecasting during COVID‐19	Relative monthly errors: July 5.7%, August 2.2%, September 11.5%, October 0.6%, November 2.7%, December 6.8%
Kuan et al.	2022	Taiwan	SARIMA‐ETS hybrid	TB intervention forecasting	SARIMA‐ETS best for short‐term, ETS best for long‐term	SARIMA‐ETS: RMSE: 0.084, MAE: 0.067, MAPE: 0.646%; ETS: RMSE: 0.079, MAE: 0.065, MAPE: 0.627%
Liao et al.	2012	Taiwan	Poisson regression	TB trend predictors	Temperature, seasonality, and aboriginal status important	RMSE: Taiwan 0.73, Hwalien 3.01, Taitung 3.86
Li et al.	2020	China	ARIMA/ARIMAX/RNN comparison	Meteorological influence on TB	ARIMAX improved prediction	Xuzhou—ARIMAX RMSE: 33.956; Nantong—ARIMAX RMSE: 25.884; Wuxi—ARIMAX RMSE: 19.026
Wang et al.	2021	India/United States	ARIMA, GRNN hybrid	COVID‐19 wave prediction	Regional model specificity observed	India: ARIMA MAPE: 10.2%, ARIMA‐GRNN MAPE: 11.7%; US: ARIMA MAPE: 19.3%, ARIMA‐GRNN MAPE: 17.3%
Wang et al.	2023	China	Deep learning EDAM	Hepatic echinococcosis detection	Deep learning EDAM outperformed radiologists	Internal set: accuracy: 0.952–0.981; external set: accuracy: 0.929–0.936
Wang et al.	2019	China	SARIMA‐NARNNX hybrid	Long‐term TB forecasting	SARIMA‐NARNNX hybrid showed better performance	Training: MAE: 2878.484, RMSE: 4468.578, MAPE: 0.038; testing: MAE: 3563.179, RMSE: 4917.829, MAPE: 0.038
Singh et al.	2023	India	ARIMA model	COVID‐19 impact on TB	COVID‐19 lockdown reduced TB detection	KPSS stationary, Ljung–Box *p* < 0.01
Zheng et al.	2015	China	ARIMA‐ARCH	TB morbidity modeling	ARIMA‐ARCH model superior to ARIMA	ARIMA RMSE: 2.58, MAE: 2.14, MAPE: 9.51%; ARIMA‐ARCH RMSE: 1.7, MAE: 1.56, MAPE: 6.85%
Moosazadeh et al.	2014	Iran	SARIMA model	TB incidence forecasting	Seasonal patterns captured	SARIMA AIC: 12.785, BIC: 7.785
Wang et al.	2017	China	ARIMA‐NAR hybrid	TB incidence prediction	ARIMA‐NAR hybrid outperformed ARIMA	Hybrid MSE: 0.1373, MAE: 0.2209, MAPE: 4.06%; ARIMA MSE: 0.2311, MAE: 0.3798, MAPE: 6.92%
Osei et al.	2023	Ghana	Interrupted Time Series	COVID‐19 impact on TB/HIV	Sharp declines observed during COVID‐19	TB case notifications: 20.5% drop in April, 32.7% drop in May
Ragonnet et al.	2017	Australia/The Netherlands	Compartmental models	Latent TB infection modeling	Two‐stage latency structure improved model fit	Rate of rapid progression (*ε*): 1.1e^−3^; Rate of reactivation (*ϕ*): 5.5e^−6^
Moosazadeh et al.	2013	Iran	SARIMA model	TB seasonality	Seasonal peaks identified	AIC: 12.6037, BIC: 3.6037
Zheng et al.	2020	China	SARIMA((2,0,2)(0,1,0)12)	TB incidence forecasting	High prediction accuracy achieved	RMSE: 0.62, MAE: 0.45, MAPE: 3.77%
Zhao et al.	2022	China	ARIMA, GM(1,1), LSTM comparison	TB prediction	LSTM outperformed ARIMA and GM(1,1)	LSTM MAE: 2676.08, MAPE: 0.0368, RMSE: 16,344.92
Wah et al.	2014	Singapore	ARIMA, SARIMA models	TB trend analysis by group	SARIMA captured TB decline among non‐residents	Non‐resident model: MSPE: 4.54, Adjusted *R* ^2^: 0.72
L'Hadj et al.	2019	Algeria	SARIMA model	PTB and EPTB trend analysis	Seasonal peaks in PTB and EPTB	SARIMA EPTB *R* ^2^: 0.6364, PTB *R* ^2^: 0.47
Orjuela‐Cañón et al.	2022	Colombia	ARIMA, NAR, LSTM, RBF models	TB incidence forecasting	ARIMA outperformed machine learning models	ARIMA RMSE: 5.89, MAE: 4.55, MAPE: 26.93%
Yun et al.	2022	China	SARIMA model	MDR‐TB trend analysis	Seasonal and spatial MDR‐TB clusters	SARIMA RMSE: 6.68, MAPE: 29.37%
Wibawa et al.	2022	Indonesia/Colombia	S‐CNN vs. CNN/MLP/LSTM	Time series forecasting	S‐CNN performed better than CNN, MLP, LSTM	S‐CNN MSE: 0.0121, MAPE: 10.00%; CNN: MAPE: 10.58%; MLP: MAPE: 11.51%; LSTM: MAPE: 11.40%

Abbreviations: GWR, geographically weighted regression; MAE, mean absolute error; MAPE, mean absolute percentage error; RMSE, root mean square error; TB, tuberculosis; S‐CNN, smoothed CNN.

### 3.2 Forecasting Accuracy by Model Type

Figure 2 compares the model‐wise forecasting performance, showing that hybrid models (e.g., ARIMA‐LSTM, SARIMA‐NNNAR, and CEEMDAN‐TCN) consistently outperformed traditional models in many settings. ARIMA and SARIMA showed higher MAPE values (∼25% and ∼17%, respectively), whereas hybrids frequently achieved MAPE below 10%, highlighting their superior predictive accuracy where data quality and sample size are adequate.

**FIGURE 2:**
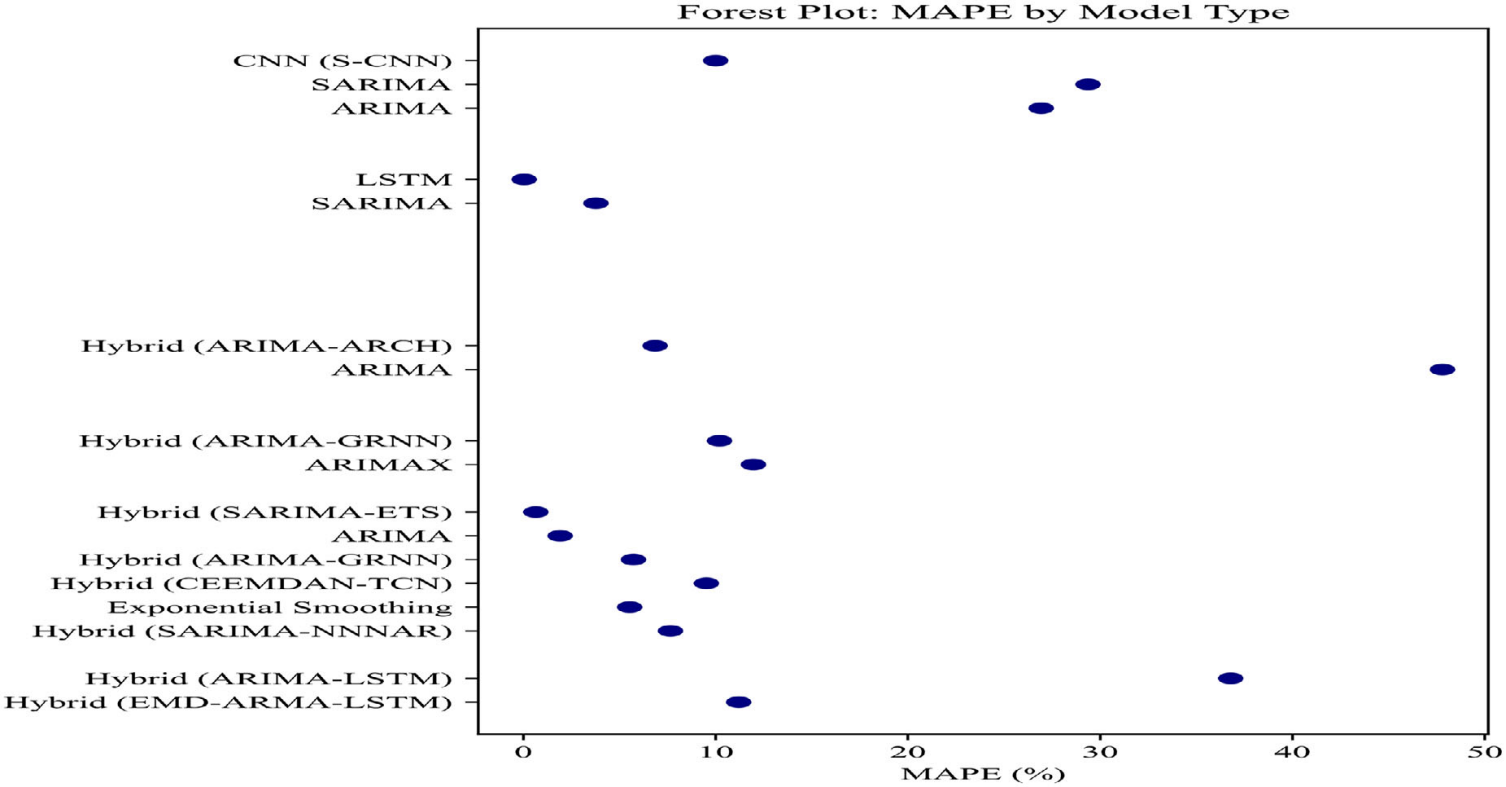
MAPE comparison between hybrid and traditional models. Forest plot of MAPE values by model type. The plot illustrates the range of MAPE values; due to extreme heterogeneity, pooled estimates are not reported. MAPE, mean absolute percentage error; S‐CNN, smoothed CNN.

### 3.3 Publication Bias Assessment

The funnel plot (Figure 3) displayed noticeable asymmetry, suggesting the presence of publication bias. Specifically, smaller studies tended to report lower MAPE values (often <5%), whereas larger studies exhibited a wider distribution of errors, ranging from approximately 8% to 48%. This imbalance implies that smaller studies with more favorable results may be overrepresented in the literature. Egger's test for funnel plot asymmetry was not statistically significant (intercept = −1.24, SE = 0.98, *p* = 0.22), but the test is underpowered given only 16 studies with available MAPE and SE. The standard error of MAPE was approximated as SD/√*n*, derived from reported confidence intervals or ranges where available.

**FIGURE 3:**
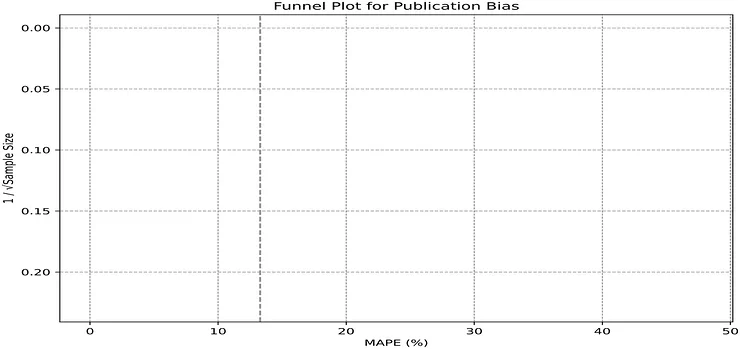
Funnel plot showing publication bias. Funnel plot assessing potential publication bias in MAPE values. The asymmetry reflects an overrepresentation of small‐sample studies reporting low errors, suggesting selective reporting in the model performance literature. MAPE, mean absolute percentage error.

### 3.4 Subgroup and Sensitivity Analysis

Subgroup analysis revealed significant geographic variation in the forecasting model performance. In studies conducted within China, single models exhibited slightly better predictive accuracy than hybrid models, with MAPE values of 8.76% and 12.95%, respectively. Conversely, studies from outside China have shown that hybrid models significantly outperform single models (MAPE: 5.42% vs. 28.24%). These findings underscore the necessity of adopting context‐specific model selection strategies tailored to regional data characteristics. However, these subgroup findings are based on small samples (12 studies within China, 4 outside China) and should be interpreted cautiously (Supplementary Table 2).

### 3.5 Risk of Bias Assessment

The methodological rigor of the included studies was systematically evaluated using the adapted Risk of Bias in Systematic Reviews (ROBIS) tool, focusing on four core domains: eligibility criteria, study selection, data collection, and synthesis. On the basis of this structured appraisal, studies were categorized as having a low, moderate, or high risk of bias. The detailed domain‐level ratings for each study are provided in Supplementary Table 3. Overall, most studies were rated as moderate to high risk, primarily due to lack of external validation (only 36% used external data), small sample sizes, and insufficient reporting of model calibration or overfitting handling. Low‐risk studies typically incorporate external validation, transparent reporting, and large datasets with relevant covariates. Moderate‐risk studies often rely on internal validation methods or have incomplete covariate integration. High‐risk studies are characterized by small sample sizes, lack of validation procedures, and limited data transparency.

### 3.6 Heterogeneity Analysis

Heterogeneity analysis revealed substantial variability among the included studies. A random‐effects meta‐analysis of MAPE values (using DerSimonian–Laird estimator) yielded a *Q*‐statistic of 6577.50 (df = 7, *p* < 0.001) and an *I* ^2^ value of 99.89%, indicating that nearly all observed variance was attributable to true differences between studies rather than random error. According to the PRISMA and Cochrane guidelines, *I* ^2^ values exceeding 75% indicate considerable heterogeneity. This exceptionally high *I* ^2^ highlights the significant methodological and clinical diversity across studies. Consequently, we did not calculate or report a pooled effect estimate; instead, we conducted a narrative synthesis.

### 3.7 Evidence‐Based Model Selection Framework

This review supports a structured approach to selecting TB forecasting models based on data characteristics and model capabilities. For small or low‐quality datasets, statistical models, like ARIMA or SARIMA, are preferred for their robustness and interpretability. For large datasets with nonlinear trends or relevant external variables, hybrid models outperform standalone approaches by combining linear and nonlinear strengths. Incorporating explainable methods ensures transparency and actionable predictions for public health decision‐making. Table 2 has been expanded to include practical implementation considerations for low‐resource settings, including minimum data requirements, software needs, and integration into surveillance systems.

**TABLE 2:** Evidence‐based model selection criteria for tuberculosis (TB) incidence forecasting in low‐ and middle‐income countries (LMICs).

Selection criterion	Scientific basis	Supporting evidence	Recommended model type	Practical considerations for LMICs
Data volume	High‐volume datasets improve model stability and reduce overfitting in deep learning models. Hybrid models perform best when trained on large time series	**Narrative synthesis shows improved MAPE in large samples (*N* > 5000) (see** Table 1)	Hybrid (e.g., ARIMA‐LSTM), Deep Learning	**Minimum 3–5 years of monthly or quarterly data; if less, prefer statistical models**
Data quality	Statistical models are less sensitive to missing or noisy data due to their deterministic structure. In contrast, ML models degrade with unstructured inputs	Higher error observed with low‐quality data (see Table 1)	ARIMA, SARIMA for low‐quality data; hybrids for structured sets	**Statistical models can handle up to 10%–15% missing data via interpolation; ML/hybrid require complete, clean series**
Seasonality and trend	SARIMA captures autoregressive and moving average patterns reflecting TB's cyclical trends	SARIMA showed consistent accuracy in seasonal datasets (see Figure S2)	SARIMA, SARIMAX hybrids	**Essential for TB, which often exhibits annual periodicity. At least four cycles needed to detect seasonality**
Nonlinear dynamics	LSTM and GRNN models capture nonlinear shifts due to epidemics or intervention changes	Hybrid models performed better during nonlinear events like COVID‐19	LSTM, GRNN, hybrid models	**Useful when outbreaks or interventions cause nonlinear shifts; requires sufficient data during such periods**
Exogenous covariates	SARIMAX integrates external variables (e.g., climate, mobility)	Limited but promising use of covariates in improving predictions	SARIMAX, ARIMAX, hybrid with external input layers	**Publicly available climate, air quality, or socioeconomic data (e.g., World Bank) can be integrated; start with simple linear terms if data sparse**
Computational constraints	ML models require higher computational resources; statistical models are lighter	SARIMA feasible on standard systems; LSTM requires GPU‐level processing	ARIMA, SARIMA, lightweight hybrid	**Open‐source software (R, Python) can be used on standard laptops for statistical models; deep learning may require cloud computing (Google Colab free tier)**
Interpretability and transparency	Statistical models provide explainable coefficients; interpretable ML methods enhance trust	Statistical and explainable ML models preferred in public health settings	ARIMA, SARIMA, hybrid + explainable AI (XAI)	**SHAP or LIME can be applied to any ML model to explain predictions to health officials**
Validation framework	Time‐series CV, rolling origin, and external test sets improve generalizability	Validation limitations found in 71% of studies (see Supplementary Table 2)	Models using robust time‐series validation	**Use rolling window or out‐of‐sample testing; if external data unavailable, at least temporally separated test set**
Regional variation	Forecasting accuracy varies across geographic and contextual settings due to differences in data structure and system heterogeneity	Subgroup analysis showed single models slightly outperformed hybrids in China, whereas hybrids outperformed single models in non‐China regions (see Supplementary Table 3)	Context‐specific: single models in structured local datasets; hybrid models in heterogeneous settings	**Pilot models on local data before scaling; consider transfer learning if neighboring region data exist**
**Data availability**	**Minimum 5 years of monthly data recommended for hybrid models**	**Most successful studies had at least 60 time points**	**Based on data volume and quality**	**LMICs can start with ARIMA/SARIMA, collect data while building capacity for hybrid models**
**Technical expertise**	**Basic statistical knowledge for ARIMA; programming skills for ML**	**Hybrid models require familiarity with both statistical and ML tools**	**Match model to available team skills**	**Free online courses and open‐source communities can help bridge skill gaps**
**Integration with health systems**	**Models should be designed with interoperability in mind**	**Few studies integrated with routine data systems**	**Prefer models that output probabilistic forecasts (e.g., prediction intervals)**	**Develop simple dashboards (R Shiny, Power BI) to visualize forecasts for decision‐makers**

Abbreviations: MAPE, mean absolute percentage error; ML, machine learning.

## 4 Discussion

This systematic review and narrative synthesis aimed to evaluate and compare the performance of statistical, ML, and hybrid models in forecasting TB incidence. The results consistently demonstrate that hybrid models often outperform traditional statistical models (e.g., ARIMA and SARIMA) and standalone ML models (e.g., LSTM and GRNN) in terms of predictive accuracy [5, 6]. Hybrid models, particularly ARIMA‐LSTM, combine the strengths of both linear statistical modeling and nonlinear ML, allowing for better handling of TB's time‐series and seasonal patterns [5, 7]. However, the extreme heterogeneity (*I* ^2^ = 99.89%) and geographic disparities observed (China vs. non‐China) indicate that no single model type is universally superior; context‐specific factors such as data quality, sample size, and epidemiological patterns heavily influence performance.

### 4.1 External Validation and Generalizability

A significant limitation is the low use of external validation in the reviewed studies, with only 36% incorporating external datasets. This affects the generalizability of the models across regions. Future research should prioritize external validation to ensure that models are robust and applicable in various settings, reducing overfitting and improving reliability [10, 11]. Incorporating diverse datasets will help test the model's accuracy in new contexts [12]. In particular, the heavy concentration of studies from China (∼70%) limits the applicability of findings to other regions with different health systems, data infrastructures, and TB epidemiology. The contrasting subgroup results (single models better in China, hybrids better elsewhere) underscore this limitation and call for more diverse geographical representation in future research.

### 4.2 Interpretability and Explainable AI (XAI)

Although hybrid models enhance forecasting accuracy, their complexity limits their interpretability. ARIMA models offer transparent coefficients, but ML techniques like LSTM are often considered “black boxes.” To address this, future work should integrate XAI techniques, such as SHAP values or attention mechanisms, to enhance transparency, ensuring that models remain trustworthy for public health decision‐making [13, 14, 15].

### 4.3 Data Quality and Exogenous Variables

The quality of the data used for training the models is crucial for accuracy. Smaller or noisy datasets often lead to higher forecasting errors, particularly in LMICs. Adopting robust data management and noise‐handling techniques will improve the performance of hybrid models, which require large high‐quality datasets for optimal forecasting [16, 17]. Fewer than 30% of the studies integrated exogenous variables, such as climatic, demographic, or socioeconomic factors. These variables can enhance model accuracy, especially in LMICs, where social determinants significantly affect TB incidence. Future research should incorporate these variables to improve predictions and inform targeted interventions [20, 21, 22, 23]. Our expanded framework (Table 2) provides practical guidance on how to identify and integrate such covariates even in data‐sparse settings, for example, by using freely available climate or satellite data.

### 4.4 Heterogeneity and Publication Bias

Substantial heterogeneity across studies, including differences in model types, data sources, and sample sizes, contributed to the variability in the results. This heterogeneity, confirmed by an *I* ^2^ of 99.89%, precluded meaningful meta‐analysis and emphasizes the need for standardized reporting and methodological consistency. Publication bias, indicated by funnel plot asymmetry and an underpowered Egger's test, suggests that smaller studies with favorable results may be overrepresented. This highlights the need for methodological consistency and transparent reporting in future studies to avoid distorting model performance [18, 19].

### 4.5 Implementation in LMICs

A major gap identified is the lack of practical implementation guidance. Most studies focus on algorithmic performance without considering real‐world constraints in LMICs, such as limited computational resources, intermittent data, and weak health information systems. To bridge this gap, we expanded the framework (Table 2) to include concrete recommendations on minimum data requirements, software options, technical expertise, and integration pathways. For instance, countries with sparse data can begin with simple ARIMA models while gradually building capacity for hybrid approaches. Furthermore, translating improved forecast accuracy (e.g., reducing MAPE from 10% to 5%) into actionable public health outcomes—such as timely resource allocation or targeted active case finding—requires close collaboration between modelers and policymakers. Future studies should explicitly discuss the policy relevance of their accuracy gains.

The key areas for future research in this domain can be summarized as follows. First, external validation is essential to ensure that models are tested on external datasets, thereby improving their generalizability. This will enhance the robustness of the models across different conditions. Secondly, the integration of exogenous variables, such as climate and socioeconomic factors, is crucial for improving forecasting accuracy. These variables can provide deeper insights into the underlying drivers of the systems being modeled. Another important direction is the use of XAI techniques, which can help improve model transparency and trust. By making the decision‐making process of AI systems more understandable, stakeholders can better interpret and rely on model predictions. Finally, large‐scale real‐time validation of models is necessary to assess their performance in dynamic, real‐world settings. This would provide continuous feedback and allow for the optimization of models under changing conditions. Additionally, researchers should report model performance using standardized metrics and include uncertainty intervals, conduct prospective studies in LMICs, and develop user‐friendly tools that can be integrated into routine surveillance systems.

## 5 Conclusion

This systematic review and narrative synthesis highlight that hybrid models often achieve higher accuracy than traditional statistical (e.g., ARIMA and SARIMA) and ML models (e.g., LSTM and GRNN) in forecasting TB incidence. Hybrid models, particularly those that integrate ARIMA with LSTM, effectively capture both linear and nonlinear trends, leading to improved predictive accuracy. However, extreme heterogeneity and contextual variations mean that model choice must be tailored to specific settings. Limitations such as the limited use of external validation, underutilization of exogenous variables, geographic concentration of studies, and challenges with model interpretability must be addressed in future research. To ensure the selection of the most appropriate model, the evidence‐based model selection framework with practical implementation considerations should be used before developing forecasting models, considering the data characteristics and specific context. Hybrid models hold great promise for improving TB forecasting accuracy, but careful consideration of validation, data quality, and model interpretability is crucial for their successful application in global TB control efforts, especially in resource‐limited LMICs.

## Author Contributions

**Dip Bahadur Singh:** Conceptualization, methodology, investigation, software, formal analysis, data curation, visualization, writing – original draft. **Bibek Giri:** writing – review and editing, validation, formal analysis, data curation, investigation. **Bipindra Pandey:** investigation, funding acquisition, writing – review and editing, visualization, validation, methodology, data curation, software, formal analysis, conceptualization. **Yashoda Dangi:** Methodology, Investigation, writing – original draft. **Sagun Kharel:** Methodology, data validation, data curation, writing – review and editing. **Roshan Kumar Mahato:** conceptualization, writing – review and editing, and supervision.

## Funding

The authors have nothing to report.

## Ethics Statement

The authors have nothing to report.

## Consent

The authors have nothing to report.

## Conflicts of Interest

The authors declare no conflicts of interest.

## Supporting information




**Supplementary Table 1**: Complete search strategy for each database.
**Supplementary Table 2**: Subgroup and sensitivity analysis (China vs. non‐China) with mean MAPE.
**Supplementary Table 3**: Meta regression analysis.

## Data Availability

All relevant data are in the article, and any query regarding the findings of this study can be obtained from the corresponding author upon request.
