# Supplementary material for: Optimizing Tuberculosis Incidence Prediction: A Systematic Review of Hybrid Modeling Approaches and Machine Learning Techniques
Source: Public Health Chall. 2026 Sep 29;5(4):e70391. doi: 10.1002/puh2.70391 (PMC13621930; doi:10.1002/puh2.70391)
Supplement: Supplementary file 1 [file PUH2-5-e70391-s001.docx]

**Optimizing Tuberculosis Incidence Prediction: A Systematic Review of Hybrid Modeling Approaches and Machine Learning Techniques**.

Dip Bahadur Singh ^1^, Bipindra Pandey^2*^, Bibek Giri^3^, Yashoda Dangi^4^, Sagun Kharel^5^, Roshan Kumar Mahato^6^

^1^Department of Health Informatics, School of Engineering, Kathmandu University, Nepal

^2^Department of Pharmacy, Madan Bhandari Academy of Health Sciences, Hetauda, Bagmati Province, Nepal

^3^Research Institute for Collaborative Development, Kathmandu, Nepal

^4^Valley College of Technical Sciences, Purbanchal University, Nepal

^5^Department of Nursing, Madan Bhandari Academy of Health Sciences, Hetauda, Bagmati Province, Nepal

^6^Faculty of Public Health, Khon Kaen University, Khon Kaen, Thailand

***Corresponding author**: Bipindra Pandey ([bipindra.pandey@mbahs.edu.np](mailto:bipindra.pandey@mbahs.edu.np))

# **Supplementary Table 1**

# **Database-Specific Search Strategy (January 1, 2013 – January 31, 2025)**

Supplementary Table 1 Search Strategy

| **Database / Source** | **Search Terms / String** | **Search Type** | **Notes** |
| --- | --- | --- | --- |
| **PubMed** | ("tuberculosis"[MeSH Terms] OR "TB"[All Fields]) AND ("forecasting"[MeSH Terms] OR "prediction"[All Fields] OR "incidence"[All Fields]) AND ("statistical model"[All Fields] OR "machine learning"[All Fields] OR "hybrid model"[All Fields] OR "ARIMA"[All Fields] OR "SARIMA"[All Fields] OR "LSTM"[All Fields] OR "GRNN"[All Fields]) AND ("MAPE"[All Fields] OR "RMSE"[All Fields] OR "accuracy"[All Fields]) | MeSH + Free Text | Adapted MeSH syntax; advanced search used |
| **Embase** | "tuberculosis" AND ("forecasting" OR "prediction" OR "incidence") AND ("statistical model" OR "machine learning" OR "hybrid model" OR "ARIMA" OR "SARIMA" OR "LSTM" OR "GRNN") AND ("MAPE" OR "RMSE" OR "accuracy") | Emtree terms + keywords | Emtree terms adapted; synonyms included |
| **Web of Science** | Topic = ("tuberculosis" OR "TB") AND ("forecasting" OR "prediction" OR "incidence") AND ("machine learning" OR "ARIMA" OR "hybrid model") AND ("MAPE" OR "RMSE" OR "accuracy") | Topic search | Advanced search mode |
| **Scopus** | TITLE-ABS-KEY ("tuberculosis" AND ("forecasting" OR "prediction") AND ("machine learning" OR "ARIMA" OR "SARIMA" OR "hybrid") AND ("MAPE" OR "RMSE" OR "accuracy")) | Title, abstract, keyword | Used filters for publication year and document type |
| **Google Scholar** | "Tuberculosis incidence prediction" AND "machine learning" OR "ARIMA" OR "hybrid model" | Free-text keyword search | Grey literature and conference abstracts |
| **Manual Reference List Review** | N/A | Citation tracking | References from included articles were manually screened for additional studies |

Summary of search terms, Boolean operators, and indexing methods used across six sources (PubMed, Embase, Web of Science, Scopus, Google Scholar, and manual reference lists) for studies published between January 1, 2013, and January 31, 2025.

# **Supplementary Table 2**

**Subgroup and Sensitivity Analysis:**

Supplementary Table 2. Comparison of Forecasting Accuracy (MAPE) Between Single and Hybrid Models by Region

| Regions | Models | Count | Mean | Std | min | 25% | 50% | 75% | max | MAPE% |
| --- | --- | --- | --- | --- | --- | --- | --- | --- | --- | --- |
| China | Hybrid | 6.0 | 12.95 | 11.83 | 5.72 | 7.04 | 8.58 | 10.78 | 36.79 | 12.95 |
|  | Single | 6.0 | 8.76 | 10.89 | 0.03 | 2.37 | 4.64 | 10.35 | 29.37 | 8.76 |
| Oher | Hybrid | 2.0 | 5.42 | 16.75 | 0.64 | 3.03 | 5.42 | 7.81 | 10.20 | 5.42 |
|  | Single | 3.0 | 28.24 | 18.93 | 10.00 | 18.46 | 26.93 | 37.36 | 47.80 | 28.24 |

Tabl 1A comparison of average MAPE values between single and hybrid models stratified by geographic region (China vs. non-China) highlights the influence of regional context on predictive performance due to the sample size

# **Supplementary Table 3**

**Meta-Regression Analysis**

Supplementary Table 3 Meta-Regression Analysis

| **Variable** | **Coefficient** | **Std. Error** | **t-Statistic** | **p-Value** | **CI Lower (95%)** | **CI Upper (95%)** |
| --- | --- | --- | --- | --- | --- | --- |
| Intercept | 31.3071 | 8.1736 | 3.8303 | 0.0314 | 5.2951 | 57.3190 |
| C(Model_Type)[T.ARIMAX] | -19.3372 | 15.6940 | -1.2321 | 0.3057 | -69.2826 | 30.6083 |
| C(Model_Type)[T.CNN (S-CNN)] | -21.2839 | 15.6930 | -1.3563 | 0.2681 | -71.2261 | 28.6583 |
| C(Model_Type)[T.Exponential Smoothing] | 28.4492 | 27.0673 | 1.0511 | 0.3704 | -57.6908 | 114.5893 |
| C(Model_Type)[T.Hybrid (ARIMA-ARCH)] | -24.3299 | 15.6851 | -1.5511 | 0.2187 | -74.2469 | 25.5871 |
| C(Model_Type)[T.Hybrid (ARIMA-GRNN)] | -23.3421 | 12.5123 | -1.8655 | 0.1590 | -63.1617 | 16.4776 |
| C(Model_Type)[T.Hybrid (ARIMA-LSTM)] | 5.4928 | 15.6940 | 0.3500 | 0.7495 | -44.4526 | 55.4383 |
| C(Model_Type)[T.Hybrid (EMD-ARMA-LSTM)] | -20.0987 | 15.6942 | -1.2806 | 0.2904 | -70.0445 | 29.8471 |
| C(Model_Type)[T.Hybrid (SARIMA-ETS)] | -18.5087 | 15.7461 | -1.1754 | 0.3246 | -68.6197 | 31.6023 |
| C(Model_Type)[T.Hybrid (SARIMA-NNNAR)] | -19.1480 | 15.4819 | -1.2368 | 0.3042 | -68.4182 | 30.1221 |
| C(Model_Type)[T.LSTM] | 160.4767 | 86.6172 | 1.8527 | 0.1610 | -115.1780 | 436.1313 |
| C(Model_Type)[T.SARIMA] | 3.9494 | 13.5898 | 0.2906 | 0.7903 | -39.2994 | 47.1982 |
| Sample_Size | -0.0001 | 0.0000 | -2.1823 | 0.1171 | -0.0002 | 0.0000 |

Table 3 Regression coefficients, p-values, and diagnostic metrics summarizing the effects of model type, sample size, and covariates on forecasting accuracy (MAPE) across included studies
